# Microbiota-mediated gut–brain communication following whey protein supplementation and resistance training: current evidence linking gastrointestinal function and cognitive health

**DOI:** 10.3389/fcimb.2026.1918695

**Published:** 2026-09-22

**Authors:** Hao Li, Jiaxin Zheng, Guanqun Zhang, Xiyue Dong, Zhigang Zhang, Shan Lu, Feng Gao

**Affiliations:** 1 Qinggong College, North China University of Science and Technology, Tangshan, Hebei, China; 2 North China University of Science and Technology, Tangshan, Hebei, China; 3 Tangshan Polytechnic University, Tangshan, Hebei, China; 4 Tianjin Academy of Fine Arts, Tianjin, China

**Keywords:** brain health, cognitive performance, gastrointestinal function, gut hormones responses, gut microbiota, gut–brain axis, resistance training, whey protein

## Abstract

Whey protein supplementation in conjunction with resistance training (RT) has emerged as a promising strategy that may modulate microbiota-mediated gut–brain communication, with potential implications for gastrointestinal (GI) function and cognitive health. Evidence from both preclinical and clinical studies suggests that whey protein supplementation and exercise, including RT, may influence gut microbial composition and function through changes in substrate availability, intestinal physiology, and host–microbe interactions. Whey protein supplementation has been associated with source-dependent changes in gut microbial communities, including alterations in the abundance of Bacteroidetes, Bifidobacterium, and butyrate-producing taxa, whereas exercise particularly aerobic exercise, with more limited evidence for RT—has been associated with greater microbial diversity and an increased abundance of short-chain fatty acids (SCFAs)-producing bacteria. Although direct evidence evaluating the combined effects of whey protein supplementation and RT remains limited, available findings suggest that their combination may influence microbial metabolic activity in addition to microbial composition. Microbiota-derived metabolites, particularly SCFAs, together with exercise- and whey protein-induced changes in gut hormone secretion, including glucagon-like peptide-1, peptide YY, and cholecystokinin, may represent plausible mechanistic links between the GI tract and the central nervous system. These pathways may contribute to the regulation of intestinal barrier integrity, neuroendocrine communication, immune responses, and neuroplasticity. Experimental evidence further suggests that whey protein supplementation combined with exercise, including RT, may contribute to reduced oxidative stress and neuroinflammation and may support brain-derived neurotrophic factor signaling; however, direct evidence linking microbiota-mediated changes to cognitive outcomes in combined intervention studies remains scarce. Nevertheless, findings remain heterogeneous because of differences in protein dosage, exercise protocols, intervention duration, study populations, and individual microbiome variability. Overall, current evidence supports the biological plausibility of microbiota-mediated gut–brain communication as a potential mechanism underlying the effects of whey protein supplementation and RT; however, well-designed human studies directly evaluating gut microbiota, gut–brain signaling, and cognitive outcomes are needed to confirm these proposed relationships.

## 1 Introduction

Whey protein supplementation and resistance training (RT) are widely recognized for their complementary roles in promoting skeletal muscle adaptation and metabolic health. More recently, increasing attention has focused on their potential interactions with the gut microbiota and gut–brain axis, extending interest beyond their established effects on muscle protein synthesis (MPS) and body composition. Emerging evidence suggests that both interventions may influence host physiology through interconnected microbial, endocrine, neural, and immune pathways, although the underlying mechanisms and their clinical relevance remain incompletely understood. The gut microbiota has significant implications for nutrient metabolism, gut hormone secretion, intestinal barrier integrity, and neurochemical synthesis, all of which can impact systemic homeostasis and cognitive functioning (Chen et al., 2021; Rusch et al., 2023). RT appears to influence gut microbial composition and function, although current evidence is considerably more limited than that available for endurance exercise. Physical activity has been demonstrated to affect gut microbial diversity and metabolite profiles, potentially influencing intestinal barrier integrity and central nervous system (CNS) signaling through microbial metabolites such as short-chain fatty acids (SCFAs) and modulation of inflammatory pathways (Dalton et al., 2019; Cataldi et al., 2022; Zhang et al., 2025). However, caution is warranted when extrapolating findings from endurance-based exercise studies to RT because these exercise modalities differ substantially in their physiological and metabolic adaptations. Despite rapid advances in these research areas, the available literature remains fragmented.

Comprehensive reviews have individually examined the impact of exercise on the microbiota–gut–brain axis, alongside explorations of probiotic and dietary interventions on cognitive health (Cataldi et al., 2022; Mishra and Mewani, 2025); conversely, other studies have addressed the implications of whole foods on metabolic processes and mental health within more generalized frameworks (Boscaini et al., 2023). By comparison, studies specifically evaluating RT-induced alterations in the gut microbiota remain relatively scarce and report heterogeneous findings, reflecting differences in study populations, training protocols, dietary control, microbiome sequencing methods, and outcome measures (Cullen et al., 2023; Wagner et al., 2024). Similarly, narrative reviews of dietary interventions have largely focused on satiety, cardiometabolic outcomes, or gut microbial changes within broader nutritional contexts, with limited attention to the combined influence of whey protein supplementation and RT on microbiota-mediated gut–brain communication (Boscaini et al., 2023; Cava et al., 2024). Emerging human and preclinical studies suggest that whey protein supplementation may modify specific microbial taxa and microbial metabolic activity; however, reported effects on SCFAs production and other microbial biomarkers remain inconsistent across studies (Cronin et al., 2018; Gao et al., 2025). Preclinical studies further suggest that whey protein supplementation may increase bacterial diversity and improve selected cognitive behaviors in animal models, providing biologically plausible mechanisms linking dietary protein, the gut microbiota, and brain function; however, translation of these findings to humans remains uncertain (Jones Severino Vasconcelos et al., 2024). Direct human evidence remains limited. Available randomized controlled trials indicate that whey protein supplementation may improve selected cognitive outcomes in some populations, whereas the addition of RT has not consistently demonstrated synergistic cognitive benefits. These findings emphasize both the limited availability of direct evidence and the need for further mechanistic and well-controlled human studies evaluating gut microbiota, gut–brain signaling, and cognitive outcomes within the same intervention (Griffen et al., 2024).

Although several reviews have examined individual components of the gut–brain axis, a comprehensive synthesis integrating the available evidence on whey protein supplementation, RT, gut microbiota, gastrointestinal (GI) physiology, and gut–brain communication is currently lacking. Importantly, the available evidence varies substantially across these domains, with considerably fewer studies directly evaluating the combined effects of whey protein supplementation and RT. Existing reviews have primarily focused on exercise-associated alterations in the gut microbiota, probiotic interventions, or the metabolic effects of dietary protein, often addressing these topics independently rather than within an integrated gut–brain framework (Cataldi et al., 2022; Boscaini et al., 2023; Cava et al., 2024; Mishra and Mewani, 2025). Consequently, it remains uncertain whether reported changes in microbial composition, microbial metabolites, or gut hormone secretion translate into coordinated physiological adaptations relevant to gut–brain communication, particularly in individuals undertaking structured RT together with whey protein supplementation. Accordingly, this narrative review aims to critically synthesize current evidence across molecular, microbial, physiological, and clinical levels while explicitly distinguishing direct evidence from mechanistic and indirect evidence. Particular attention is given to areas of consistency, sources of heterogeneity, and important knowledge gaps requiring further investigation.

Specifically, this review first examines the available evidence regarding the independent effects of whey protein supplementation and RT on gut microbial composition and function, before evaluating studies investigating their combined effects. It subsequently explores whether microbiota-associated changes, including alterations in microbial metabolites, gut hormone secretion, and GI physiology, may contribute to gut–brain communication and cognitive outcomes. Where direct evidence is unavailable, mechanistic inferences from preclinical and related human studies are discussed separately. In addition, the review critically evaluates methodological factors that contribute to heterogeneity across studies, including differences in whey protein dosage, RT protocols, intervention duration, participant characteristics, dietary control, microbiome profiling techniques, and outcome assessment. Overall, available evidence suggests that whey protein supplementation and RT each influence host physiology through multiple biological pathways; however, direct evidence examining their combined effects on microbiota-mediated gut–brain communication remains limited. By integrating findings across nutritional, microbial, physiological, and neurobiological domains, this review aims to identify consistent mechanistic patterns, reconcile conflicting findings, distinguish established evidence from biologically plausible hypotheses, and highlight priorities for future research. Rather than providing clinical recommendations, this review seeks to establish an evidence-based framework for interpreting current findings and identifying the key questions that should be addressed in future well-controlled human studies.

## 2 Literature search strategy and study selection

This article was conducted as a narrative review aimed at integrating current evidence regarding the potential interactions between whey protein supplementation, RT, gut microbiota, GI function, and microbiota-mediated gut–brain communication. Unlike systematic reviews, which primarily focus on predefined quantitative synthesis, narrative reviews allow critical interpretation and integration of heterogeneous evidence across different experimental models, intervention designs, and biological domains. Nevertheless, a structured literature identification approach was applied to enhance transparency and reproducibility. A comprehensive literature search was performed in the electronic databases PubMed/MEDLINE, Web of Science, Scopus, and Google Scholar from database inception to May 2026. The search strategy combined keywords and Medical Subject Headings (MeSH) related to four main domains (Chen et al., 2021): whey protein supplementation (Rusch et al., 2023), RT/exercise interventions (Dalton et al., 2019), gut microbiota and GI physiology, and (Zhang et al., 2025) gut–brain axis and cognitive outcomes. The following combinations of search terms were used: (“whey protein” OR “whey protein supplementation” OR “dietary protein”) AND (“resistance training” OR “resistance exercise” OR “strength training”) AND (“gut microbiota” OR “intestinal microbiome” OR “microbial metabolites” OR “short-chain fatty acids”) AND (“gut–brain axis” OR “cognition” OR “cognitive function” OR “brain health” OR “neuroinflammation” OR “brain-derived neurotrophic factor (BDNF)”). The term “RT” was used throughout the manuscript as the primary term, while related terms such as “resistance exercise” and “strength training” were included only as search keywords to maximize literature retrieval.

Studies were considered eligible when they investigated at least one of the following aspects: (i) the effects of whey protein supplementation on gut microbial composition, microbial metabolites, GI function, or gut hormone responses; (ii) the effects of RT on gut microbiota-related outcomes or gut–brain-associated pathways; (iii) combined whey protein supplementation and RT interventions; or (iv) mechanistic links connecting microbial alterations with neuroendocrine, inflammatory, or cognitive outcomes. Both human and preclinical studies were considered to provide a comprehensive mechanistic perspective, while evidence from different models was interpreted according to the strength and translational relevance of each study design. Studies were excluded if they were unrelated to whey protein or resistance exercise, focused exclusively on non-protein dietary interventions, lacked relevance to gut microbiota or gut–brain communication, were conference abstracts, non-peer-reviewed sources, editorials, or articles without sufficient methodological information. In addition, studies with substantial methodological concerns, including unclear experimental design, absence of appropriate comparator groups, incomplete reporting of outcomes, or insufficient methodological transparency, were excluded from interpretation of clinical efficacy. Throughout the review process, particular attention was given to distinguishing direct evidence from combined whey protein plus RT interventions from indirect evidence derived from independent whey protein studies, exercise studies, or preclinical models. To further strengthen methodological rigor, the methodological quality and risk of bias of included studies were considered according to study design. Although formal risk-of-bias scoring tools were not applied because this was a narrative review rather than a systematic review, methodological limitations and potential sources of bias were critically considered during evidence interpretation. Randomized controlled trials were critically evaluated based on key methodological domains, including randomization procedures, allocation concealment, blinding where applicable, intervention adherence, completeness of outcome reporting, and potential confounding factors. Observational studies were interpreted with consideration of participant selection, adjustment for relevant confounders, exposure assessment, and outcome measurement reliability. Preclinical studies were evaluated according to the appropriateness of experimental models, intervention characterization, sample size considerations, and reproducibility of reported findings. Studies with major methodological limitations, insufficient intervention details, unclear outcome assessment, lack of appropriate controls, or inadequate reporting were not considered as primary evidence for strong mechanistic or clinical conclusions. Instead, such studies were considered only when they provided preliminary insights or hypothesis-generating evidence. Because this was a narrative review, evidence synthesis was based on predefined relevance criteria, methodological robustness, biological plausibility, and contribution to the central research question rather than quantitative pooling. In addition, exercise modality was considered when interpreting findings, with specific emphasis placed on differentiating RT-specific evidence from endurance or mixed RT interventions.

## 3 Whey protein and exercise: physiological and metabolic overview

Whey protein, a high-quality dairy-derived protein, is characterized by rapid digestibility and a high content of essential amino acids, particularly branched-chain amino acids such as leucine, isoleucine, and valine. Leucine acts as an important nutritional signal for skeletal MPS, primarily through activation of the mechanistic target of rapamycin complex 1 (mTORC1) pathway (Phillips, 2014; Devries and Phillips, 2015). Although these anabolic effects represent the most established biological actions of whey protein, increasing evidence suggests that whey protein may also influence broader physiological networks, including metabolic regulation, GI function, and host–microbe interactions. RT induces mechanical and metabolic stimuli that activate adaptive signaling pathways involved in muscle remodeling, including mTORC1, AMP-activated protein kinase, and insulin-like growth factor-1 pathways (Egan and Zierath, 2013). The combination of RT and whey protein supplementation is widely used to support muscle adaptation, with evidence indicating greater improvements in muscle protein turnover and functional outcomes compared with either intervention alone in many populations (Ji et al., 2025). However, the magnitude of these effects varies according to factors such as age, training status, dietary background, protein dose, and intervention duration. Beyond skeletal muscle adaptation, whey protein may influence metabolic regulation through effects on insulin secretion and glucose homeostasis. Whey-derived amino acids, particularly leucine, together with incretin-related responses involving glucagon-like peptide-1 (GLP-1) and glucose-dependent insulinotropic polypeptide (GIP) (Nilsson et al., 2004; Jakubowicz and Froy, 2013), may contribute to postprandial metabolic regulation. These endocrine responses are also relevant to gut physiology because gut-derived hormones represent potential communication pathways linking nutrient sensing, metabolism, and the gut–brain axis. From a GI perspective, whey protein is rapidly digested in the small intestine, resulting in increased availability of amino acids and bioactive peptides that may interact with intestinal processes (Jahan-Mihan et al., 2011; Sah et al., 2016). In parallel, RT may influence GI physiology through alterations in gut motility, intestinal permeability, and systemic metabolic responses. Unlike endurance exercise, RT represents a distinct physiological stimulus characterized by intermittent high-intensity muscle contractions, repeated increases in intra-abdominal pressure, transient hemodynamic fluctuations, and unique neuroendocrine responses. These factors may influence GI physiology differently by modifying splanchnic blood flow distribution, mechanical stress on the GI tract, autonomic regulation, and stress hormone signaling. In addition, the anabolic and inflammatory adaptations induced by RT, including changes in myokine release and muscle–organ communication, may create a different systemic environment for host–microbiota interactions compared with prolonged aerobic exercise. Therefore, findings from endurance-based exercise studies should not be directly extrapolated to RT, and RT-specific investigations are required to determine its independent effects on gut microbial composition, microbial metabolites, and gut–brain communication. Nevertheless, evidence directly examining RT-specific effects on GI function and gut microbiota remains limited, and a substantial proportion of available data is derived from endurance or mixed exercise models. Emerging evidence suggests that both whey protein supplementation and exercise may influence inflammatory and oxidative stress-related pathways (Alhebshi et al., 2021; Prokopidis et al., 2023). Whey-derived bioactive peptides have demonstrated antioxidant and immunomodulatory properties in experimental models, whereas RT may contribute to improved inflammatory regulation through repeated physiological adaptation. Nevertheless, the extent to which these effects are mediated through microbiota-dependent mechanisms remains unclear. Importantly, potential links between whey protein, RT, and brain health are discussed in subsequent sections focusing on microbiota-mediated gut–brain communication, where microbial metabolites, gut hormones, immune signaling, and neurotrophic pathways are considered within the context of available evidence (**Figure 1**).

**Figure 1:**
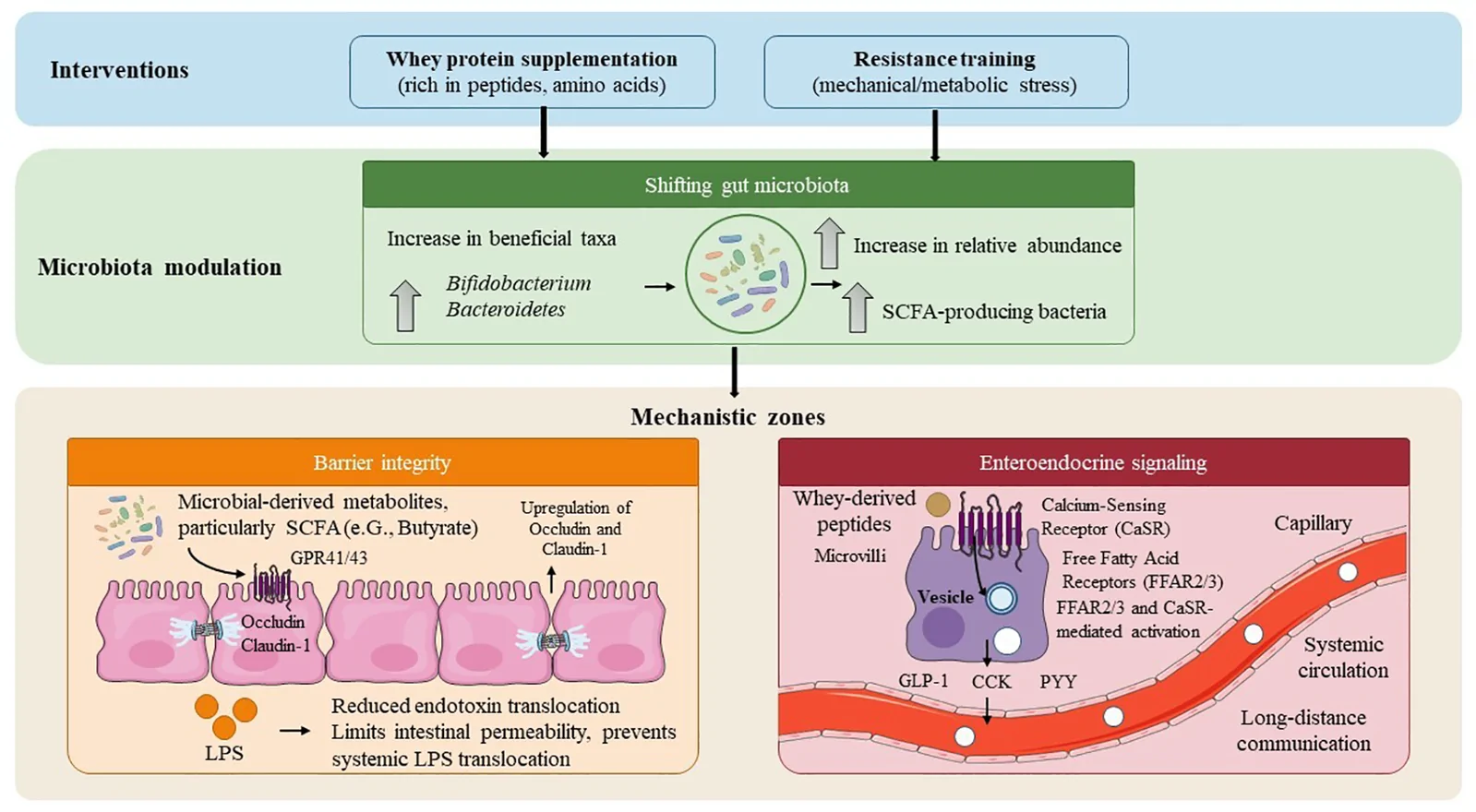
Mechanistic adaptations within the intestinal microenvironment following combined whey protein and resistance training. The figure outlines how the synergy between dietary whey-derived bioactives and resistance exercise-induced stimuli modulates the gut ecosystem. Key local physiological outputs include the reinforcement of the epithelial barrier, through upregulated tight junction proteins (e.g., Occludin), and the triggered release of satiety and regulatory hormones (e.g., GLP-1, PYY, CCK) via enteroendocrine receptor activation (CaSR/FFAR). This figure represents a proposed mechanistic model based primarily on preclinical evidence and indirect findings; these pathways have not been fully validated as causal mechanisms in humans undergoing combined whey protein supplementation and resistance training.

## 4 Modulation of gut microbiota, GI function and gut hormone responses by whey protein and exercise

The gut microbiota is increasingly recognized as an important contributor to host metabolism, immune regulation, and neurophysiological processes through interactions with GI function and gut-derived hormonal signaling. Both dietary protein intake and RT interventions may influence microbial composition, diversity, and metabolic activity through changes in nutrient availability, intestinal transit, host immune responses, and microbial metabolite production. Among nutritional interventions, whey protein supplementation has been associated with alterations in selected microbial taxa and microbial metabolites, although findings remain heterogeneous depending on protein source, dosage, dietary background, and population characteristics. Similarly, exercise-induced changes in the gut microbiota have been reported, but evidence specific to RT remains limited compared with endurance-based exercise modalities (Clark and Mach, 2016; Mailing et al., 2019). Therefore, distinguishing the independent effects of whey protein supplementation and RT is essential before interpreting potential combined effects. Beyond taxonomic alterations, increasing evidence suggests that microbiota-derived metabolites, including SCFAs, secondary bile acids, indole derivatives, and neurotransmitter-related precursors, may participate in communication between the gut and host systems by influencing intestinal barrier function, immune signaling, and endocrine pathways. Through these mechanisms, microbial metabolites may contribute to gut hormone secretion, intestinal barrier regulation, immune modulation, and gut–brain signaling; however, the extent to which these pathways mediate the effects of combined whey protein supplementation and RT remains to be established (Cryan et al., 2019; Fan and Pedersen, 2021).

### 4.1 Effects of whey protein on gut microbial composition and diversity

Whey protein supplementation may influence gut microbial composition through its high digestibility, amino acid profile, and generation of bioactive peptides during GI digestion. Although most whey-derived amino acids are absorbed in the small intestine, a fraction of undigested protein and bioactive peptides may reach the colon, where they can interact with resident microbial communities and contribute to changes in microbial metabolism (Sánchez-Moya et al., 2017). Available experimental studies suggest that whey protein supplementation may be associated with alterations in specific microbial taxa, including changes involving potentially beneficial genera such as *Lactobacillus* and *Bifidobacterium*; however, these findings are not uniform across populations and intervention protocols (McAllan et al., 2014). Microbial fermentation of protein-derived substrates can generate metabolites including SCFAs, indole derivatives, branched-chain fatty acids, and secondary bile acids, which may participate in regulating intestinal epithelial function, immune responses, and enteroendocrine signaling. Indole metabolites derived from tryptophan metabolism may activate aryl hydrocarbon receptor-dependent pathways involved in mucosal immune regulation and intestinal barrier maintenance (Agus et al., 2018). Nevertheless, the magnitude and physiological relevance of these metabolite changes following whey protein supplementation remain dependent on dietary context, baseline microbiota composition, and individual metabolic characteristics. Preclinical and mechanistic studies further suggest that whey-derived bioactive peptides may possess antimicrobial and immunomodulatory properties, although direct evidence demonstrating selective microbial modulation in humans remains limited (Wen et al., 2023). Therefore, potential effects of whey protein on microbial diversity and ecological balance should be interpreted cautiously, as responses may vary according to protein dose, dietary background, and host characteristics. Dose magnitude represents an important determinant of GI tolerance and potential microbiome-related responses to whey protein supplementation. In most exercise and aging-related interventions, moderate whey protein doses typically range from approximately 20–40 g per serving or 40–80 g/day depending on the population, training status, and total dietary protein intake (Morton et al., 2018). These doses are generally well tolerated and are commonly used to support muscle adaptation during RT without consistent evidence of adverse GI effects (Cermak et al., 2012). In contrast, higher supplemental protein intakes, particularly when total protein consumption exceeds approximately 2.0–2.5 g/kg/day or when large single boluses are consumed, may increase the likelihood of GI symptoms in susceptible individuals (Antonio et al., 2016). Excessive protein availability may also promote greater colonic proteolytic fermentation, potentially increasing metabolites such as ammonia and phenolic compounds (Windey et al., 2012). However, the relationship between whey dose, microbial alterations, and GI outcomes remains influenced by dietary context, habitual fiber intake, baseline microbiota composition, and individual tolerance. Consistent with this dose-dependent concept, excessive protein intake or specific dietary conditions may increase microbial proteolytic fermentation and production of metabolites such as ammonia and phenolic compounds, highlighting that the effects of whey protein on the microbiome are context-dependent rather than universally beneficial (Diether and Willing, 2019). Beyond direct microbial effects, whey protein may interact with gut hormone signaling, creating a bidirectional relationship between nutrient sensing, microbial metabolism, and enteroendocrine responses. Microbiota-derived fermentation products may influence enteroendocrine hormone secretion, including GLP-1, peptide YY (PYY), and GIP, through pathways involving free fatty acid receptors (FFAR2 and FFAR3) expressed on enteroendocrine cells (Tolhurst et al., 2012). These pathways provide a biologically plausible link between whey protein intake, microbial metabolites, and gut–brain communication; however, direct evidence connecting these processes to cognitive outcomes remains insufficient. Some studies suggest that whey protein may support intestinal barrier function through modulation of tight junction-related pathways and inflammatory responses, although these findings are primarily derived from specific experimental settings and require further validation in diverse human populations (Arbizu et al., 2020). Overall, current evidence indicates that whey protein supplementation may modulate gut microbial ecology and related metabolic pathways, but the direction and magnitude of these effects remain heterogeneous across studies. Importantly, baseline microbiome composition, sex, age, dietary patterns, and methodological differences in microbiome assessment may contribute substantially to interindividual variability, supporting the need for personalized approaches in microbiome-targeted nutrition (Kolodziejczyk et al., 2019).

### 4.2 Exercise-induced alterations in gut microbiota

RT may generate distinct gut microbial and GI responses compared with endurance exercise because of differences in physiological stress patterns and systemic adaptations. Unlike prolonged aerobic exercise, RT consists of repeated short-duration, high-intensity contractions that induce transient increases in intra-abdominal pressure, mechanical loading of the trunk musculature, intermittent alterations in splanchnic blood flow, and unique autonomic and neuroendocrine responses. These characteristics may influence intestinal motility, barrier function, immune signaling, and microbial substrate availability in ways that differ from endurance-based exercise. Moreover, RT promotes muscle-derived endocrine signaling through myokines and anabolic pathways, which may contribute to muscle–gut communication and potentially shape host–microbiota interactions. However, whether these RT-specific physiological features translate into distinct and reproducible microbiome signatures remains unclear and requires direct comparative investigations. Aerobic exercise and physically active lifestyles have frequently been associated with greater microbial diversity and alterations in microbial taxa involved in SCFA production, including *Faecalibacterium prausnitzii* and *Roseburia species* (Clarke et al., 2014; Estaki et al., 2016; Allen et al., 2018); however, these observations should not be directly generalized to RT because resistance-based adaptations may involve different physiological and metabolic stimuli. These observations suggest that exercise-related changes in microbial metabolism may contribute to intestinal and systemic adaptations; however, consistent microbiota signatures have not been established across all populations. Microbial metabolites, particularly SCFAs such as butyrate, represent potential mediators linking exercise-induced microbial changes with host physiology. SCFAs may contribute to intestinal barrier regulation, immune modulation, and metabolic homeostasis through effects on epithelial integrity, inflammatory pathways, and immune cell function (Tan et al., 2014; Campbell and Turner, 2018; Scheiman et al., 2019). Nevertheless, whether these metabolite alterations directly result from RT or represent a broader consequence of increased physical activity remains unclear. Mechanistically, exercise may influence gut microbial ecosystems through multiple interconnected pathways, including alterations in intestinal transit, immune surveillance, metabolic substrate availability, and host endocrine signaling. Exercise-related modulation of mucosal immunity, including changes in immunoglobulin A responses and inflammatory mediators, has been proposed as one pathway through which microbial communities may be shaped (Campbell and Turner, 2018). However, many mechanistic insights are derived from observational or experimental exercise models, and their translation to long-term RT interventions requires further investigation. Exercise may also influence bile acid metabolism, potentially affecting microbial ecological niches and host signaling pathways involving receptors such as farnesoid X receptor and Takeda G protein-coupled receptor 5. These pathways may contribute to regulation of glucose metabolism, energy balance, inflammation, and gut hormone secretion, suggesting a possible microbiota–bile acid–host signaling axis (Campbell et al., 2017). However, direct evidence demonstrating that RT-induced microbiota alterations regulate these pathways in humans remains scarce. Importantly, exercise-induced microbial responses are not uniformly beneficial and may vary according to exercise dose. Moderate training is generally associated with favorable metabolic and microbial adaptations, whereas excessive training loads or prolonged high-intensity exercise may increase intestinal permeability and GI disturbances in susceptible individuals (Mach and Fuster-Botella, 2017). This dose-dependent relationship highlights the importance of considering exercise prescription when interpreting microbiome-related outcomes. Exercise may additionally influence gut-derived hormones, including GLP-1, PYY, and ghrelin, which regulate appetite, GI motility, and metabolic signaling. These hormonal changes may indirectly affect microbial ecology by modifying nutrient availability and intestinal environmental conditions; however, direct links between exercise-induced hormonal responses, microbiota alterations, and gut–brain outcomes remain insufficiently characterized. Overall, current evidence suggests that exercise can influence gut microbial composition and function, but RT-specific microbiome adaptations remain an emerging area of research. Future controlled studies directly comparing RT with other exercise modalities and incorporating microbiome, metabolomic, and clinical outcomes are required to determine whether RT produces unique microbiota-mediated effects relevant to gut–brain communication.

### 4.3 Combined effects of whey protein supplementation and exercise: implications for microbial function, gut hormone responses, and GI outcomes

The combined effects of whey protein supplementation and RT on gut microbial function represent an emerging but incompletely characterized area of research. Although both interventions individually influence host metabolism and may interact with microbial pathways, direct evidence demonstrating that whey protein plus RT produces coordinated microbiota-mediated adaptations remains limited. Current evidence is derived from a heterogeneous range of human interventions, athlete studies, and preclinical models, with substantial variability in protein type, exercise modality, intervention duration, microbiome assessment methods, and measured outcomes.

### 4.4 Human evidence from combined protein supplementation and RT interventions

Human studies incorporating protein supplementation with structured resistance-based exercise interventions suggest that whey protein may contribute to selective microbial and GI adaptations; however, findings remain inconsistent. In elite athletes undergoing RT, an 8-week randomized trial comparing whey protein concentrate with plant-based protein sources demonstrated that protein source influenced specific microbial taxa and predicted metabolic pathways, although overall microbial diversity remained unchanged (Kroplewski et al., 2026) (**Table 1**). Whey supplementation was associated with alterations in microbial composition, including increased relative abundance of selected bacterial groups; however, the study did not directly evaluate gut–brain outcomes, and functional predictions were based on sequencing-derived inference rather than metabolomic validation. Similarly, studies in older adults combining whey protein supplementation with resistance-based exercise interventions have reported variable microbiome responses. In pre-frail and frail individuals receiving whey or rice protein alongside RT, both groups achieved comparable functional outcomes, while whey supplementation was associated with a higher frequency of GI symptoms, highlighting that potential benefits of protein supplementation may be accompanied by individual tolerability differences (Jadczak et al., 2021). Importantly, studies comparing different protein sources during RT suggest that microbial responses may not be exclusive to whey protein. For example, in healthy adults undergoing RT, plant-derived protein supplementation produced greater increases in microbial diversity and SCFA-associated taxa compared with animal-derived protein supplementation, indicating that protein source, dietary matrix, and accompanying nutrients may strongly influence microbiome outcomes (Govindan et al., 2026). These findings suggest that the interaction between RT and dietary protein is likely context-dependent rather than representing a uniform whey-specific microbiome effect.

**Table 1:** Effects of whey protein supplementation and exercise on gut microbiota, gut hormones, and gastrointestinal function.

Population/Model	Exercise modality	Study design	Duration	Intervention	Microbiome method	Direct outcomes measured	Main findings	Main limitations	Study
Elite football players (n=45)	Resistance training (RT)	Randomized controlled parallel trial	8 weeks	Resistance training plus 30 g/day whey protein concentrate (WPC), pea protein isolate (PPI), rice protein isolate (RPI), or mixed plant protein	Oxford Nanopore 16S rRNA sequencing (V3–V8); functional prediction using MACADAM/STAMP	Microbial alpha diversity, taxonomic composition, predicted metabolic pathways	Alpha diversity remained unchanged; WPC increased *Bacteroidetes* abundance; RPI enriched predicted butyrate-producing pathways; protein source differentially modulated microbial composition and metabolic potential	Small sample size; predicted functional pathways were not experimentally validated; findings limited to elite athletes	(Kroplewski et al., 2026)
Trained athletes with vs. without GI symptoms (n=71)	No structured exercise intervention (athletic population)	Observational comparison with pre–post intervention	3 weeks	Multi-ingredient fermented whey supplement	16S rRNA gene sequencing	Gut microbiota composition, gastrointestinal symptoms, physical and mental wellbeing	Baseline microbiota did not differ between groups; supplementation increased *Bifidobacterium*, reduced severe gastrointestinal symptoms, and improved physical wellbeing	No placebo/control group; short intervention; relatively small sample	(Wardenaar et al., 2024)
Obese exercising rats	Endurance exercise (treadmill)	Experimental animal study	Not reported	Egg- or whey-protein diets with or without inulin combined with treadmill exercise	Gut microbiota profiling with predicted metagenomics and plasma metabolomics	Gut microbiota composition, predicted microbial pathways, plasma metabolites, gut hormones, glucose metabolism	Whey/inulin improved energy balance, increased CCK, PYY, and proglucagon expression, reduced insulin and leptin, and favorably altered gut microbiota and butyrate-related metabolism	Animal model; limited clinical translatability	(Avirineni et al., 2022)
Sedentary adults	Endurance exercise	Prospective intervention study	Short-term intervention	Exercise with or without daily whey protein supplementation	Shotgun metagenomics and metabolomics	Gut microbial composition, gut virome diversity, microbial function, metabolomic profile	Exercise induced modest microbiota changes; whey supplementation significantly altered gut virome diversity; improved body composition occurred independently of major microbiota changes	Short intervention; relatively small sample; sedentary adults only	(Cronin et al., 2018)
Endurance athletes (n=24)	Endurance exercise	Randomized, double-blind, placebo-controlled pilot trial	10 weeks	Whey isolate plus beef hydrolysate vs. maltodextrin	16S rRNA sequencing; fecal SCFA analysis	Taxonomic composition, SCFAs, fecal pH, ammonia, oxidative stress	Increased *Bacteroidetes* abundance but reduced *Roseburia*, *Blautia*, and *Bifidobacterium longum*; no changes in SCFAs or oxidative stress	Pilot study with small sample size	(Moreno-Pérez et al., 2018)
Recreational runners (n=13)	Endurance exercise	Single-blind crossover clinical trial	Acute (single exercise session)	0.15 vs. 0.4 g/kg whey protein consumed 60∑min before a 10-km run	Not assessed	Gastrointestinal symptoms, gut fullness, blood glucose, perceived exertion	Protein dose did not affect most gastrointestinal symptoms or performance; higher dose increased bloating only	No microbiome analysis; small sample size; acute intervention	(Shaw et al., 2026)
Older adults (n=70)	Resistance training	Randomized controlled pilot trial	6 months	Multicomponent exercise plus whey protein or rice protein (2 × 20 g/day)	Not assessed	Physical performance, frailty, cognition, gastrointestinal symptoms	Similar functional improvements between protein groups; whey supplementation caused approximately twofold more gastrointestinal symptoms than rice protein	No microbiome assessment; pilot study	(Jadczak et al., 2021)
Older adults	No exercise intervention	Comparative supplementation study	12 weeks	Essential amino acids vs. whey protein	Illumina 16S rRNA gene amplicon sequencing	Gut microbiota composition, IL-18, 6-minute walk test	Gut microbiota remained stable in both groups; essential amino acids reduced IL-18 and improved physical performance compared with whey protein	No placebo control; relatively short intervention	(Azhar et al., 2024)
Healthy adults (n=24)	Resistance training	Randomized controlled intervention	12 weeks	Lentil protein vs. animal protein vs. placebo	16S rRNA sequencing; GC-FID for SCFA analysis	Microbial diversity, SCFA concentrations, predicted functional pathways	Plant protein increased microbial diversity, SCFA-producing bacteria, butyrate, acetate, and pathways associated with gut barrier integrity	Small sample size; functional pathways were computationally predicted	(Govindan et al., 2026)
Rats	Endurance exercise (treadmill)	Experimental animal study	6 weeks	Fermented milk containing whey protein, *Bifidobacterium animalis* BB12, and pomegranate followed by acute exercise	Intestinal microbiota profiling	Gut microbiota composition, intestinal motility, villus morphology, inflammatory markers	Supplementation restored *Lactobacillus/Clostridium* balance, preserved villus structure, improved intestinal motility, and protected against exercise-induced intestinal injury	Animal model; combined intervention precluded isolation of whey protein-specific effects	(Chaves et al., 2018)

CCK, cholecystokinin; GC-FID, gas chromatography–flame ionization detection; GI, gastrointestinal; IL-18, interleukin-18; PPI, pea protein isolate; RPI, rice protein isolate; SCFAs, short-chain fatty acids; STAMP, Statistical Analysis of Metagenomic Profiles; WPC, whey protein concentrate.

### 4.5 Exercise populations receiving whey supplementation

Evidence from physically active populations provides additional insights but should be interpreted cautiously because many studies involve endurance or mixed exercise rather than RT-specific protocols. In trained athletes with GI complaints, fermented whey supplementation was associated with improvements in selected GI symptoms and increased abundance of *Bifidobacterium*; however, baseline microbiota differences were not strongly associated with symptom severity, suggesting complex relationships between microbial composition and GI function (Wardenaar et al., 2024). Conversely, a short-term intervention in previously inactive individuals demonstrated that exercise training alone induced modest microbiome alterations, whereas the addition of whey protein supplementation produced broader changes in gut virome diversity without substantial changes in overall bacterial diversity (Cronin et al., 2018). These findings suggest that microbial adaptation may require prolonged behavioral exposure and may extend beyond conventional bacterial diversity metrics. Protein supplementation studies in endurance athletes have also reported inconsistent microbial outcomes. A randomized pilot study found that protein supplementation altered selected bacterial taxa but did not significantly modify fermentation markers, SCFA concentrations, or oxidative stress parameters (Moreno-Pérez et al., 2018). Similarly, pre-exercise whey protein intake influenced GI symptoms during running, with moderate doses increasing bloating in some participants, emphasizing the importance of individualized responses and exercise context (Shaw et al., 2026).

### 4.6 Preclinical and mechanistic evidence

Animal and *ex vivo* studies provide mechanistic insights into potential pathways linking whey protein, exercise, and microbial metabolism, although their translational relevance requires cautious interpretation. In obese male rats, whey protein combined with treadmill exercise and dietary fibers altered gut microbial composition, plasma metabolites, energy regulation, and gut hormone responses, including changes involving PYY, cholecystokinin, and proglucagon signaling (Avirineni et al., 2022). However, the contribution of whey protein, exercise, and fiber independently could not be fully separated. Similarly, fermented whey-containing interventions in exercised rodents preserved intestinal morphology, microbial composition, and GI motility following high-intensity exercise-induced intestinal stress, although improvements in physical performance were not observed (Chaves et al., 2018).

### 4.7 Critical synthesis

Collectively, current evidence indicates that whey protein supplementation combined with exercise may influence microbial composition, gut hormone signaling, and GI function; however, the evidence supporting a specific synergistic effect of whey protein plus RT on microbiota-mediated gut–brain communication remains preliminary. Most available studies do not simultaneously assess microbial composition, microbial metabolites, gut hormones, and cognitive outcomes within the same intervention. Furthermore, differences in exercise modality, protein dose, dietary background, microbiome sequencing approaches, and participant characteristics contribute substantially to variability between studies. In particular, the predominance of endurance-based evidence represents an important limitation when attempting to define RT-specific microbiota adaptations. Therefore, current findings support a biologically plausible interaction between whey protein, RT, and gut microbial pathways rather than a confirmed causal model. Future controlled human trials integrating microbiome profiling, metabolomics, GI assessments, and neurocognitive outcomes are required to determine whether combined whey protein and RT interventions generate clinically meaningful gut–brain effects (**Figure 2**).

**Figure 2:**
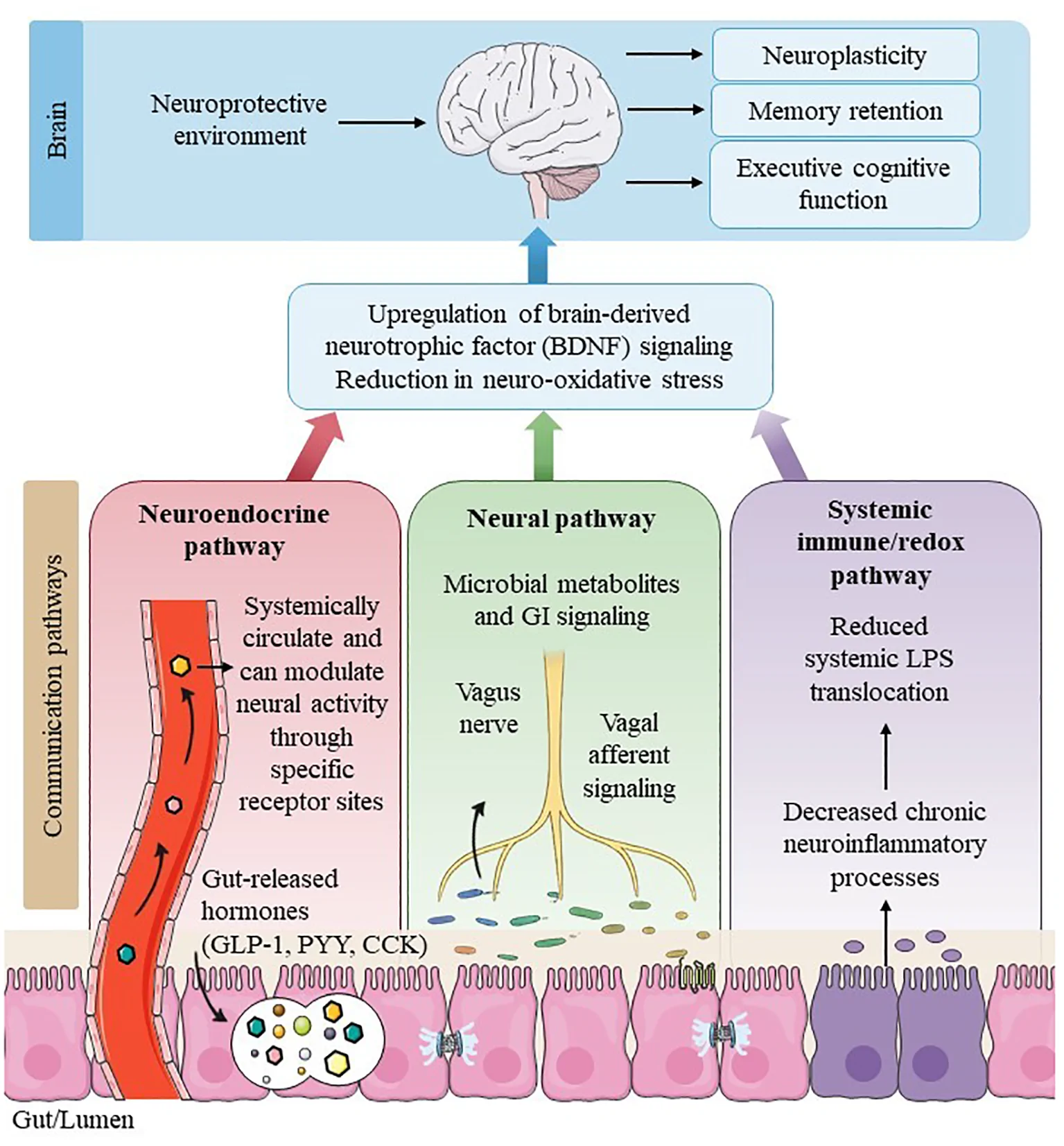
The integrated microbiota-mediated gut–brain axis and cognitive outcomes. This conceptual model describes the systemic transition from intestinal adaptations to central nervous system (CNS) health. GI-derived signals communicate with the brain through three primary conduits: neuroendocrine, vagal afferent, and immune-redox signaling pathways. These integrated signals converge to support a neuroprotective environment, characterized by increased Brain-Derived Neurotrophic Factor (BDNF) signaling and attenuated neuroinflammation, which collectively facilitate improvements in executive function and neuroplasticity. This figure represents a proposed mechanistic framework based on preclinical evidence and indirect human observations and should not be interpreted as a fully validated causal pathway demonstrating microbiota-mediated cognitive effects in human whey protein plus resistance training interventions.

## 5 Microbiota-mediated gut–brain communication in response to whey protein and RT

### 5.1 Mechanistic pathways linking gut and brain

The gut–brain axis represents a complex bidirectional communication network involving neural, endocrine, immune, and metabolic pathways. Key components include the vagus nerve, the hypothalamic–pituitary–adrenal (HPA) axis, intestinal immune signaling, and microbial-derived metabolites such as SCFAs (Cryan et al., 2019). Current evidence suggests that gut microbiota may contribute to gut–brain communication through the production of bioactive metabolites, including SCFAs, secondary bile acids, indole derivatives, and neurotransmitter-related compounds. However, direct evidence demonstrating that these microbial signals causally mediate cognitive adaptations in humans remains unavailable, particularly following combined whey protein supplementation and RT. Microbial metabolites may influence enteroendocrine signaling, vagal afferent activity, immune responses, and blood–brain barrier function, thereby providing potential pathways through which intestinal changes could affect neurophysiological processes (Cryan et al., 2019; Morais et al., 2021). Nevertheless, most mechanistic evidence has been generated from preclinical models or studies examining exercise or dietary interventions independently, rather than controlled trials directly evaluating whey protein combined with RT. Importantly, human studies have not yet established whether microbiota alterations function as causal mediators of cognitive responses following whey protein supplementation and RT. Most available evidence remains associative, linking microbial profiles or metabolites with physiological and cognitive outcomes without demonstrating temporal or mechanistic mediation. Whey protein supplementation may theoretically influence gut–brain signaling by modifying amino acid availability, including tryptophan metabolism, which is involved in serotonin biosynthesis and neurobehavioral regulation. However, whether whey-induced alterations in microbial tryptophan metabolism translate into measurable cognitive benefits in humans remains unclear. Similarly, RT has been associated with changes in systemic metabolism, inflammatory status, and neurotrophic signaling; however, the contribution of microbiota-mediated mechanisms to these adaptations requires further clarification. In contrast to endurance exercise, RT introduces distinct physiological demands that may differentially affect GI function and host–microbiota relationships. High-intensity resistance training involves repeated rises in intra-abdominal pressure, especially during multi-joint movements and Valsalva maneuvers, along with short-lived redistribution of blood flow from the splanchnic circulation toward active skeletal muscles (Costa et al., 2017; Hawley et al., 2025). These hemodynamic shifts can temporarily modify intestinal perfusion, the availability of oxygen to the epithelium, and mucosal barrier integrity, thereby shaping luminal microbial activity and metabolite generation. RT also produces a neuroendocrine environment that differs from endurance exercise, including immediate increases in growth hormone, testosterone, catecholamines, and cortisol, which may affect intestinal permeability, immune cell trafficking, substrate use, and host–microbiota signaling (Mailing et al., 2019; Hawley et al., 2025). Moreover, the dominance of mechanical loading and anaerobic metabolism during RT may alter microbial substrate availability by affecting amino acid turnover, lactate metabolism, and protein use, which could result in microbiome adaptations that differ from those seen after endurance exercise. However, these mechanistic propositions largely rely on indirect evidence, and only a small number of controlled human studies have directly described microbiome composition, microbial metabolites, or gut barrier function after RT combined with whey protein supplementation. As a result, microbiota changes reported after endurance exercise should not be directly applied to RT until they are confirmed by appropriately designed intervention studies. Microbial metabolism of dietary amino acids can generate neuroactive compounds, including indole derivatives and metabolites involved in neurotransmitter pathways, which may participate in host–microbe communication and influence mood, stress responses, and cognitive processes (Agus et al., 2018). These pathways represent biologically plausible mechanisms; however, no controlled human study has yet demonstrated that microbiota-derived signaling directly mediates the cognitive or neurophysiological effects of combined whey protein supplementation and RT. Experimental studies suggest that SCFAs, particularly butyrate, may influence BDNF expression, microglial function, and hippocampal neurogenesis (Allen et al., 2018; Dalile et al., 2019). However, the extent to which exercise-induced microbial alterations contribute to BDNF regulation in humans remains uncertain. Whey-derived bioactive peptides may also influence intestinal barrier-related processes, potentially affecting systemic inflammation and neuroimmune signaling. Therefore, whey protein supplementation and RT may interact with multiple biological pathways relevant to gut–brain communication, but current evidence supports a mechanistically plausible model rather than a confirmed causal pathway. Overall, the gut microbiota represents a potential biological interface connecting dietary protein intake, exercise adaptations, and brain-related outcomes. Current evidence therefore supports a mechanistically plausible model rather than a validated causal pathway linking combined whey protein supplementation and RT to cognitive adaptations through microbiota-mediated gut–brain communication.

### 5.2 Role of microbiota in neuroendocrine signaling

The gut microbiota has been proposed as an important contributor to neuroendocrine communication through the production of microbial metabolites, modulation of enteroendocrine activity, and regulation of host immune and stress-related pathways. Certain bacterial taxa, including *Lactobacillus* and *Bifidobacterium*, have been reported to participate in the metabolism of neuroactive compounds, including gamma-aminobutyric acid and precursors involved in serotonin and dopamine pathway (Clarke et al., 2014). However, the extent to which these microbial processes directly influence human neurocognitive outcomes remains uncertain, particularly in response to whey protein supplementation combined with RT. Beyond neurotransmitter-related mechanisms, gut microbiota may influence neuroendocrine signaling through microbial metabolites such as SCFAs. SCFAs can activate FFAR2/3 expressed on enteroendocrine cells, potentially affecting the secretion of gut-derived hormones including GLP-1, PYY, and serotonin, which are involved in appetite regulation, metabolic control, and communication with the CNS (Tolhurst et al., 2012). Although these pathways provide a biologically plausible mechanism linking microbial activity with host neuroendocrine responses, direct evidence demonstrating that whey protein–induced or RT-induced microbiota alterations regulate these hormonal pathways in humans remains limited. RT interventions have been associated with changes in microbial diversity and metabolic profiles; however, most available evidence originates from aerobic or mixed exercise models, and RT-specific effects on microbiota-mediated neuroendocrine signaling remain insufficiently characterized. Exercise-associated microbial adaptations have also been proposed to influence stress-related pathways, including HPA axis activity and emotional regulation. Some experimental studies suggest that microbiota-derived signals may affect stress responsiveness through immune and endocrine mechanisms (Monda et al., 2017). However, these findings are primarily derived from mechanistic or preclinical investigations, and their relevance to combined whey protein and RT interventions requires validation in controlled human studies. Microbial metabolites may further contribute to gut–brain communication by influencing peripheral signaling pathways, immune responses, and potentially neuroplasticity-related processes. Experimental evidence from germ-free animal models indicates that the absence of microbiota can impair microglial maturation, whereas SCFA supplementation may partially restore microglial function, highlighting a potential microbiota–immune–brain pathway (Erny et al., 2015). While these findings provide important mechanistic insights, extrapolation from germ-free models to human nutritional and RT interventions should be performed cautiously. Overall, microbiota-mediated neuroendocrine signaling represents a biologically plausible mechanism through which dietary protein and RT may influence systemic physiology and brain-related outcomes. However, current evidence remains insufficient to establish that combined whey protein supplementation and RT improve cognition through microbiota-dependent neuroendocrine pathways, and causal relationships in humans have yet to be demonstrated.

### 5.3 Inflammation, oxidative stress, and neural communication

Chronic inflammation and oxidative stress serve as significant impediments to the integrity of the gut-brain axis and the efficacy of neural communication. The elevation of intestinal permeability, commonly referred to as “leaky gut,” facilitates the translocation of lipopolysaccharides (LPS), which in turn instigates systemic inflammation and neuroinflammatory responses (Köhler et al., 2016). However, the extent to which exercise- or nutrition-induced changes in intestinal permeability translate into altered brain inflammation or cognitive outcomes remains incompletely established in humans. Disruption of intestinal barrier integrity facilitates the systemic dissemination of microbial-associated molecular patterns, including LPS, which activate Toll-like receptor signaling pathways and promote microglial activation, neuroinflammation, and cognitive dysfunction (Fung et al., 2017). These mechanisms provide a biologically plausible link between intestinal microbial activity and neuroimmune regulation; nevertheless, much of the current evidence originates from preclinical studies and cannot be directly extrapolated to whey protein plus RT interventions. Whey protein encompasses bioactive constituents such as lactoferrin and cysteine-rich peptides that promote the synthesis of glutathione, consequently diminishing oxidative stress and inflammatory responses (Marshall, 2004). Although these properties suggest potential anti-inflammatory effects, the contribution of whey-derived bioactive compounds to microbiota-mediated gut–brain communication remains insufficiently defined. RT further alleviates inflammatory signaling by downregulating pro-inflammatory cytokines and bolstering antioxidant defenses. Exercise-induced increases in SCFAs-producing bacteria may additionally suppress nuclear factor-kappa B signaling and promote regulatory T-cell differentiation, thereby attenuating systemic and neuroinflammation (Tan et al., 2014). However, whether these adaptations are specific to RT, rather than representing general consequences of physical activity, remains unclear. Moreover, both exercise and whey protein may enhance intestinal barrier integrity, thereby restricting endotoxin translocation and maintaining neural homeostasis. Enhanced expression of tight junction proteins, including zonula occludens-1, occludin, and claudins, may represent an important mechanism through which these interventions preserve gut barrier integrity and limit endotoxemia (Ghosh et al., 2020). Nevertheless, direct evidence demonstrating that whey protein combined with RT enhances gut barrier function and subsequently modifies neuroinflammatory pathways in humans is currently limited. Reducing inflammatory burden and oxidative stress may theoretically support neurotransmission, neuronal resilience, and neuroplasticity. Therefore, modulation of microbiota-associated inflammatory and redox pathways represents a biologically plausible mechanism linking whey protein supplementation and RT with gut–brain communication; however, this framework should currently be regarded as a mechanistic hypothesis rather than an established causal pathway because direct evidence demonstrating microbiota-mediated effects on cognitive outcomes in humans remains lacking.

## 6 Effects of whey protein and exercise on brain health

### 6.1 Evidence from preclinical and clinical studies

Evidence linking whey protein supplementation and exercise, particularly RT, with cognitive outcomes through microbiota-mediated gut–brain mechanisms remains limited and heterogeneous. Available studies have primarily investigated indirect pathways, including oxidative stress regulation, neurotrophic signaling, inflammation, and metabolic adaptation, rather than simultaneously measuring gut microbiota alterations and cognitive outcomes.

Preclinical investigations provide mechanistic insights into potential interactions between dietary protein, exercise, and brain function. In a murine model of scopolamine-induced cognitive impairment, whey protein hydrolysate combined with treadmill exercise improved memory-related performance and was associated with reduced oxidative stress markers, enhanced cholinergic signaling, increased BDNF expression, and reduced apoptosis-related pathways (Chang et al., 2023) (**Table 2**). The study also reported alterations in gut microbial composition, suggesting a potential microbiota-related mechanism; however, because the model was based on pharmacologically induced cognitive impairment and animal experimentation, the findings cannot be directly extrapolated to human whey protein and RT interventions. Human evidence remains considerably less conclusive. A randomized crossover study in elite basketball athletes examined the effects of different protein contents in supplements consumed following endurance exercise on cerebral oxygenation and exercise performance (Ho et al., 2018). Although higher protein intake was associated with altered cerebral oxygen saturation and improved exercise capacity, the study did not evaluate gut microbiota, gut-derived metabolites, or cognitive outcomes, and therefore provides indirect evidence rather than direct support for microbiota-mediated gut–brain communication. Similarly, studies investigating whey protein supplementation combined with structured exercise interventions have reported improvements in systemic physiological outcomes, but many have not included cognitive or microbiome-related endpoints. For example, reductions in cardiac stress biomarkers following RT and whey protein supplementation may indicate broader cardiometabolic adaptations; however, these outcomes have limited relevance to microbiota-mediated cognitive pathways and are therefore outside the primary scope of this review. Overall, current evidence suggests that whey protein and RT may influence biological processes relevant to brain health, including oxidative balance, inflammation, and neurotrophic signaling. Nevertheless, direct human studies simultaneously assessing whey protein supplementation, different exercise modalities (particularly RT), gut microbiota composition, microbial metabolites, and cognitive outcomes are lacking. Therefore, current interpretations should be considered mechanistic hypotheses requiring confirmation through well-controlled clinical investigations.

**Table 2:** Whey protein supplementation and resistance training: Effects on brain health and cognitive function.

Study & population	Exercise modality	Study design	Duration	Intervention	Microbiome method	Direct outcomes measured	Main findings	Main limitations	Study
Mice with scopolamine-induced cognitive deficit	Endurance exercise (treadmill)	Experimental animal study	Not reported	Whey protein hydrolysate (100 or 200 mg/kg), treadmill exercise, or combined intervention	Gut microbiota profiling (method not specified)	Cognitive performance, oxidative stress markers (ROS, MDA), acetylcholine metabolism, tau phosphorylation, BDNF expression, apoptosis-related markers (Bax/Bcl-2), MAPK signaling, gut microbiota changes	Whey protein hydrolysate and treadmill exercise improved cognitive performance, reduced oxidative stress, increased acetylcholine and BDNF expression, inhibited tau hyperphosphorylation and apoptosis, modulated MAPK signaling, and induced favorable gut microbiota alterations	Animal model; microbiome methodology insufficiently described; limited clinical translation	(Chang et al., 2023)
15 Division 1 collegiate basketball players (18–20 y)	Endurance exercise (cycling)	Randomized counterbalanced crossover trial	Acute intervention (single exercise session with 2-h recovery period)	High-protein whey supplement (36% of total calories) vs. low-protein carbohydrate supplement (12% of total calories) after exercise	Not assessed	Cerebral oxygen saturation, cerebral blood perfusion (total hemoglobin), glucose response, insulin response, exercise endurance	High-protein supplementation enhanced cerebral oxygenation, reduced cerebral blood perfusion demand, improved glucose and insulin responses, and increased cycling time by approximately 16%	Small sample size; acute study design; no direct assessment of cognitive outcomes or long-term adaptation	(Ho et al., 2018)
40 sedentary men (60–65 y)	Resistance training	Randomized counterbalanced crossover trial	6 weeks	Resistance training, whey protein supplementation, combined exercise + whey protein, or control	Not assessed	Serum atrial natriuretic peptide (ANP), brain natriuretic peptide (BNP), and galectin-3 levels	Resistance training combined with whey protein supplementation reduced ANP, BNP, and galectin-3 levels, suggesting improved cardiovascular adaptations	Small sample size; non-randomized design; no direct cognitive outcomes; no microbiome assessment	(Ho et al., 2018)

ANP, atrial natriuretic peptide; BDNF, brain-derived neurotrophic factor; BNP, brain natriuretic peptide; MAPK, mitogen-activated protein kinase; MDA, malondialdehyde; ROS, reactive oxygen species.

## 7 Cognitive outcomes following whey protein supplementation and RT

Direct evidence demonstrating that whey protein supplementation combined with exercise, particularly RT, improves cognitive function through microbiota-mediated gut–brain pathways remains lacking. Available human studies have primarily identified associations between microbial alterations, inflammatory or metabolic markers, and cognitive-related outcomes; however, no clinical trial has formally demonstrated microbiota-mediated cognitive effects through causal approaches such as mediation analysis. Therefore, current evidence should be interpreted as hypothesis-generating rather than demonstrating a causal microbiota-dependent pathway.

### 7.1 Human evidence from whey protein and RT interventions

A randomized controlled trial investigated the independent and combined effects of RT and whey protein supplementation on cognitive function in 36 older males (mean age 67 ± 4 years) (Griffen et al., 2024) (**Table 3**). Participants received whey protein (50 g/day) or maltodextrin and were allocated to exercise or non-exercise groups for 12 weeks. Whey protein supplementation improved executive function compared with control, whereas RT alone reduced inflammatory markers, including tumor necrosis factor alpha and interleukin-6, without significant cognitive improvement. Importantly, the study did not identify synergistic cognitive effects between exercise and protein supplementation and did not evaluate gut microbiota-related mechanisms. Importantly, because neither gut microbiota composition, microbial metabolites, nor gut-derived biomarkers were assessed, the observed cognitive improvements cannot be attributed to microbiota-mediated gut–brain communication, and no causal inference regarding the role of the microbiome can be drawn from these findings. Similarly, a 12-month randomized trial examined a multicomponent intervention consisting of exercise combined with whey protein, calcium, and vitamin D in men undergoing androgen deprivation therapy for prostate cancer (Mundell et al., 2022). Despite high supplementation adherence, the intervention did not significantly improve cognitive outcomes compared with standard care. The absence of cognitive benefits in this population further emphasizes the heterogeneity of responses and the influence of clinical context, adherence, and baseline characteristics. These findings further indicate that participant characteristics, underlying disease status, baseline cognitive function, and intervention duration may substantially influence cognitive responsiveness, thereby limiting generalization across different populations. Another investigation assessed a multi-ingredient supplement containing whey protein, omega-3 fatty acids, creatine, vitamin D, and calcium combined with RT and high-intensity interval training in sedentary older men (Bell et al., 2019). Cognitive improvements were observed after the exercise phase; however, these effects were associated with omega-3-related biomarkers rather than whey protein-specific mechanisms. Because the intervention involved multiple bioactive components, the independent contribution of whey protein or RT to cognitive changes cannot be determined. Accordingly, the observed cognitive improvements cannot be specifically attributed to whey protein supplementation, RT, or microbiota-mediated mechanisms because of the multifactorial intervention design. Studies comparing different protein sources during RT have also reported cognitive outcomes. In older adults undergoing RT, bovine colostrum and whey protein supplementation produced comparable improvements in cognitive performance alongside physical adaptations (Duff et al., 2014). However, these studies did not examine microbiota composition or gut-derived signaling pathways, limiting their relevance to microbiota-mediated gut–brain communication. Collectively, these intervention studies suggest that cognitive improvements may accompany RT-based nutritional interventions; however, current human evidence does not identify gut microbiota as the mechanistic mediator underlying these effects.

**Table 3:** Effects of whey protein supplementation on sleep, cognitive function, and performance in trained populations.

Population & sample size	Exercise modality	Study design	Duration	Intervention	Microbiome method	Direct outcomes measured	Main findings	Main limitations	Study
36 older men, 67 ± 4 y	Resistance training	Randomized, double-blind, placebo-controlled trial (secondary analysis)	12 weeks	Resistance exercise (2 sessions/week), whey protein isolate (2 × 25 g/day), combined intervention, or control (maltodextrin)	Not assessed	Cognitive function (CANTAB battery), executive function, global cognition, inflammatory markers (TNF-α, IL-6), cortisol, insulin sensitivity	Whey protein supplementation improved executive function compared with placebo and showed a trend toward improved global cognitive function. Resistance exercise reduced systemic inflammatory markers but did not independently improve cognition. No synergistic effect between exercise and whey protein was observed	Small sample size; secondary analysis; older male population only; no direct mechanistic assessment of gut–brain pathways	(Griffen et al., 2024)
70 men treated with ADT	Resistance training + weight-bearing exercise	Two-arm randomized controlled trial (secondary analysis)	12 months	Multicomponent resistance and weight-bearing exercise plus whey protein (25 g/day), calcium (1200 mg/day), and vitamin D (2000 IU/day) versus usual care	Not assessed	Cognitive function (CogState battery, Trail Making Test, Rey Auditory Verbal Learning Test, Digit Span)	Exercise combined with whey protein, calcium, and vitamin D supplementation did not significantly improve cognitive outcomes compared with usual care	Disease-specific population; adherence to exercise was below recommended levels; cognitive decline may be influenced by cancer treatment-related factors	(Mundell et al., 2022)
49 sedentary men, 73 ± 6 y	Mixed exercise (RT + HIIT)	Randomized controlled trial	20 weeks (8 weeks supplementation + 12 weeks exercise phase)	Multi-ingredient supplement containing whey protein (30∑g), omega-3 fatty acids, creatine, vitamin D, and calcium with or without resistance exercise and high-intensity interval training	Not assessed	Montreal Cognitive Assessment (MoCA), Rey Auditory Verbal Learning Test, executive function, reaction time, omega-3 index	Resistance exercise combined with supplementation improved MoCA scores, memory performance, reaction time, and composite cognitive function. Cognitive improvements correlated with increased omega-3 index and reduced arachidonic acid/eicosapentaenoic acid ratio	Multi-ingredient intervention prevents attribution of effects specifically to whey protein; limited generalizability to other populations	(Bell et al., 2019)
40 adults, 59 ± 6 y	Resistance training	Randomized double-blind controlled trial	8 weeks	Resistance training combined with bovine colostrum (60 g/day) or whey protein complex (38∑g protein/day)	Not assessed	Cognitive function questionnaire, muscle strength, body composition, IGF-1, CRP, bone turnover marker (Ntx)	Both colostrum and whey protein groups improved cognitive scores, muscle strength, and lean tissue mass. Colostrum produced greater improvements in leg press strength and reduced bone resorption compared with whey protein	Cognitive assessment was questionnaire-based; small sample size; colostrum rather than whey protein was the primary intervention	(Duff et al., 2014)
17 healthy, trained males	No exercise intervention	Randomized placebo-controlled crossover trial	Acute intervention (3 experimental nights)	Pre-sleep nutritional supplement containing carbohydrate, whey protein, tryptophan, theanine, and 5′AMP at two doses versus placebo	Not assessed	Sleep architecture (polysomnography), cognitive function, postural sway, next-morning cycling performance	Nutritional supplementation had minimal effects on sleep, cognitive function, postural sway, or physical performance. No meaningful improvements in cognition were observed	Small sample; acute intervention; multi-ingredient formulation prevents isolation of whey protein effects	(Morrison et al., 2025)
19 trained participants (females n = 11)	No exercise intervention	Double-blind randomized crossover trial	3 consecutive nights	Pre-sleep α-lactalbumin supplementation (40∑g) versus control	Not assessed	Sleep architecture (dry EEG), plasma tryptophan, mood, recovery, reaction time, attention, cognitive performance	α-Lactalbumin increased plasma tryptophan, increased non-REM stage 2 sleep, reduced REM sleep duration, and improved morning reaction time and attention-related cognitive performance	Small sample size; short-term supplementation; performed under controlled conditions rather than habitual environments	(Barnard et al., 2025)
16 trained women, 27 ± 7 y	No exercise intervention (post-competition recovery study)	Randomized double-blind three-arm crossover trial	Acute recovery period (24∑h after simulated competition)	α-Lactalbumin whey protein beverage versus whey placebo or water control after evening competition	Not assessed	Sleep parameters (polysomnography), cognitive assessments, physical recovery performance	α-Lactalbumin preserved non-REM stage 2 sleep after evening competition and improved subsequent physical recovery performance	Acute study; female athletes only; no direct cognitive improvement reported	(Miles et al., 2021)

ADT, androgen deprivation therapy; A-LAC, α-lactalbumin; CANTAB, Cambridge Neuropsychological Test Automated Battery; CRP, C-reactive protein; EEG, electroencephalography; HbA1c, glycated hemoglobin; IGF-1, insulin-like growth factor-1; IL-6, interleukin-6; MoCA, Montreal Cognitive Assessment; Ntx, urinary N-telopeptide; PUFA, polyunsaturated fatty acids; TNF-α, tumor necrosis factor-alpha.

### 7.2 Indirect evidence from sleep and neurobehavioral studies

Some studies have investigated whey-derived components, particularly tryptophan-enriched whey fractions such as alpha-lactalbumin, in relation to sleep and cognitive performance. Presleep supplementation containing whey protein-derived components did not significantly improve sleep, cognition, or next-day performance in trained individuals (Morrison et al., 2025). Conversely, alpha-lactalbumin supplementation increased plasma tryptophan availability and influenced sleep architecture, with associated improvements in selected cognitive tasks (Barnard et al., 2025). Similar findings were reported in female athletes following evening exercise, where alpha-lactalbumin improved aspects of sleep recovery and subsequent performance (Miles et al., 2021). However, these studies evaluated direct nutritional effects of whey-derived components on sleep-related and cognitive outcomes rather than microbiota-mediated gut–brain pathways. Therefore, their findings cannot be interpreted as evidence that gut microbial alterations mediate neurobehavioral responses following whey protein supplementation. Although these findings suggest potential relationships between whey-derived amino acids, sleep regulation, and cognitive function, they provide evidence for nutritional and neurophysiological effects rather than microbiota-mediated mechanisms. Furthermore, sleep-related effects may involve tryptophan availability, neurotransmitter regulation, and other host physiological pathways independently of gut microbial modulation.

### 7.3 Critical synthesis

Collectively, current human evidence suggests that whey protein supplementation and RT may influence selected cognitive-related outcomes in specific populations; however, the magnitude and consistency of these effects remain heterogeneous across intervention protocols and participant characteristics. Importantly, current clinical studies have not established that these cognitive outcomes are mediated by gut microbiota alterations. A major limitation of current human studies is the lack of integrated intervention designs simultaneously assessing microbiome composition, microbial metabolites, GI biomarkers, inflammatory profiles, and standardized cognitive outcomes. Consequently, it remains unclear whether cognitive changes observed after whey protein supplementation and RT reflect microbiota-mediated effects or alternative adaptations, including improved nutritional status, reduced systemic inflammation, enhanced recovery, or exercise-related neuroplastic responses. Therefore, current evidence supports an association between whey protein supplementation, RT, and cognitive-related outcomes but does not establish a causal microbiota-dependent gut–brain pathway. Future randomized controlled trials should integrate longitudinal microbiome profiling, microbial metabolomics, GI function, inflammatory biomarkers, and validated cognitive assessments within the same cohorts to determine whether microbiota alterations represent causal mediators or correlated responses to combined nutritional and exercise interventions.

## 8 Factors influencing outcomes

### 8.1 Protein source, dose, and timing

The effects of whey protein supplementation on gut microbiota, GI function, and potential gut–brain-related outcomes may be influenced by multiple interacting factors, including protein source, dosage, timing of intake, dietary background, and individual microbiome characteristics. Importantly, available evidence remains heterogeneous, with microbial responses varying according to study population, intervention duration, baseline microbiota composition, and analytical approaches. Different protein sources may produce distinct effects on microbial composition and metabolic activity. Whey protein has been associated with alterations in specific bacterial taxa, including changes in Bacteroidetes and other microbial groups involved in protein and carbohydrate fermentation. However, these responses are not consistently observed across studies and should not be interpreted as universal microbiome-modulating effects. In contrast, plant-derived proteins are frequently associated with increased microbial diversity and enrichment of SCFA-producing taxa, potentially due to differences in fiber content, amino acid composition, and accompanying bioactive compounds. Such comparisons highlight that microbial outcomes are likely determined not only by protein type but also by the broader dietary context. Protein dose represents another important determinant of tolerability and microbial responses. Moderate whey protein intake is generally well tolerated in most exercise and clinical settings, whereas higher protein loads may increase the delivery of undigested substrates to the colon and promote proteolytic fermentation, potentially resulting in GI symptoms such as bloating or altered metabolite production. However, the threshold at which these effects occur remains unclear and may differ according to habitual protein intake, gut microbial composition, and individual metabolic characteristics. The timing of whey protein ingestion relative to exercise may also influence physiological responses by affecting amino acid availability, muscle adaptation, and GI tolerance. Although pre- and post-exercise protein consumption strategies have been widely investigated for muscle-related outcomes, their specific influence on microbiota-mediated gut–brain communication remains insufficiently explored. Moreover, combining whey protein with prebiotics, probiotics, or other nutritional components may modify microbial responses; however, the contribution of each individual component is difficult to isolate in multi-ingredient interventions. Overall, protein source, dose, and timing represent important modulators of the biological response to whey protein supplementation. Nevertheless, current evidence does not support a uniform microbiota-mediated effect, and future studies should integrate dietary assessment, microbiome profiling, metabolomic analyses, and gut–brain-related outcomes to determine how these factors influence individual responsiveness.

### 8.2 Exercise type, intensity, and duration

Exercise characteristics, including modality, intensity, and duration, represent important determinants of microbial and physiological responses to whey protein supplementation. However, current evidence does not support assuming equivalent effects across different exercise modalities. RT, endurance exercise, and mixed training programs induce distinct physiological adaptations, which may differentially influence gut microbiota composition, GI function, inflammatory signaling, and metabolic regulation. Evidence from endurance-based interventions suggests that prolonged aerobic training may promote microbial diversity and increase SCFAs-producing taxa; however, these findings cannot be directly extrapolated to RT populations because RT induces different mechanical, metabolic, and hormonal adaptations. Current RT-specific microbiome studies remain relatively limited and frequently involve small sample sizes and heterogeneous protocols, restricting definitive conclusions regarding microbiota-mediated effects. Exercise duration also appears to influence microbial adaptation. Short-term interventions generally produce modest or inconsistent microbiome changes, whereas longer training periods may provide sufficient time for more stable microbial and metabolic adaptations. Conversely, excessive exercise intensity or inadequate recovery may transiently impair intestinal barrier function, increase GI discomfort, and promote inflammatory responses. Whether whey protein supplementation can consistently counteract these exercise-induced alterations remains unclear and may depend on training load, dietary context, and individual susceptibility. Exercise intensity may additionally influence gut hormone responses, energy metabolism, and gut–brain signaling through changes in microbial metabolites, inflammation, and neuroendocrine pathways. Nevertheless, direct evidence linking specific exercise parameters with microbiota-mediated cognitive outcomes remains scarce. Future studies should therefore compare RT and endurance modalities using standardized protocols and integrated assessment of microbiota, metabolomics, gut hormones, and brain-related outcomes.

### 8.3 Individual variability

Individual characteristics, including age, training status, health condition, habitual diet, and baseline microbiota composition, represent major sources of variability in responses to whey protein supplementation and RT interventions. Interindividual differences in microbial ecology may influence the magnitude and direction of microbiome-related adaptations, highlighting the importance of personalized approaches. Sex represents another important but insufficiently investigated determinant of response to whey protein supplementation and RT. The majority of available exercise and nutritional intervention studies, particularly those involving RT and protein supplementation, have been conducted predominantly in male participants, limiting the generalizability of current findings to women. Biological sex may influence gut microbiota composition, intestinal barrier function, immune regulation, and neuroinflammatory responses through sex hormone–dependent mechanisms. Estrogens have been reported to modulate epithelial integrity, microbial diversity, and inflammatory signaling, whereas androgens may influence immune responses, metabolic regulation, and host–microbiota interactions. Consequently, differences in circulating estrogen and androgen levels across the lifespan, including menopause and aging-related hormonal changes, may contribute to variability in GI and cognitive responses following combined whey protein and RT interventions. Future studies should therefore incorporate sex-balanced designs and consider hormonal status when evaluating microbiota-related adaptations. Older adults may exhibit altered GI function, reduced microbial resilience, and different physiological responses compared with younger populations. However, improvements in physical function or cognitive-related outcomes do not necessarily correspond with measurable microbiome alterations, suggesting that multiple biological pathways may contribute to intervention effects. Training status also appears to influence responsiveness. Elite athletes often display distinct baseline microbial profiles associated with long-term training and dietary patterns, whereas sedentary individuals may demonstrate limited microbial changes following short-term interventions. Importantly, microbial characteristics observed in highly trained populations should not be assumed to represent responses in clinical or aging populations. Health status further modifies intervention outcomes. Individuals with metabolic disorders, cancer, or GI complaints may respond differently due to altered inflammatory states, medication use, dietary limitations, and differences in exercise adherence. Fermented whey products or combined nutritional strategies may provide potential benefits in selected populations; however, their specific contribution to microbiota-mediated gut–brain communication requires further investigation. Overall, variability in host characteristics represents a critical determinant of response to whey protein and RT interventions. Future clinical trials should incorporate baseline microbiome profiling, sex-specific analyses, hormonal status assessment, dietary evaluation, and standardized exercise protocols to determine how individual characteristics influence responses to whey protein and RT interventions and to identify potential predictors of benefit.

## 9 Clinical implications and practical applications

Current evidence supports whey protein supplementation combined with RT as an effective strategy for improving muscle-related outcomes and supporting adequate protein intake, with potential benefits for metabolic regulation and GI function. However, clinical translation of microbiota-mediated gut–brain mechanisms remains premature, as human evidence does not yet establish that microbiome alterations mediate cognitive effects of whey protein plus RT. Although whey protein and RT are widely used nutritional and exercise strategies, the extent to which their combined effects influence cognitive outcomes through gut microbial pathways has not yet been established in controlled human studies. From a practical perspective, whey protein can represent a useful strategy to increase protein intake and support muscle-related adaptations, particularly in populations with increased protein requirements, including older adults and individuals undergoing exercise-based interventions. However, potential effects on gut microbiota composition, gut hormone responses, or cognitive outcomes should be considered as emerging possibilities rather than established clinical benefits. Individual factors, including dietary background, baseline microbiota characteristics, GI tolerance, and health status, should be considered when designing supplementation strategies. Moderate whey protein intake is generally well tolerated in most populations, although GI responses may vary substantially between individuals. In practical terms, moderate whey protein doses commonly used in RT interventions are generally considered safe and may support adequate protein intake and GI tolerance when appropriately individualized. However, there is currently insufficient evidence to recommend whey protein combined with RT as a microbiota-targeted cognitive intervention or as a specific strategy to modify gut–brain communication. Therefore, gradual dose adjustment and monitoring of tolerance may be appropriate, particularly among individuals with pre-existing GI symptoms or altered digestive function. The combination of whey protein with probiotics, prebiotics, or fermented products represents an area of growing interest; however, the independent contribution and clinical relevance of these combined approaches require further validation. Current evidence is insufficient to determine whether such combinations produce additive microbiome-mediated cognitive benefits beyond the established effects of exercise and adequate nutrition. Exercise prescription should also be individualized according to age, health status, training experience, and functional capacity. Although RT is strongly supported for improving muscle mass and physical function, its specific contribution to microbiota-mediated gut–brain communication remains insufficiently characterized. Furthermore, many current findings regarding exercise and cognitive health are derived from broader exercise modalities rather than RT-specific interventions. From a neurocognitive perspective, preliminary studies suggest that whey protein-derived amino acids and exercise-induced physiological adaptations may influence cognitive-related outcomes through metabolic, inflammatory, and neuroendocrine pathways. Nevertheless, consistent improvements in cognition have not been demonstrated, and direct evidence connecting whey protein, RT, gut microbiota alterations, and cognitive performance remains limited. Therefore, current evidence supports the use of whey protein and RT as components of multidomain health strategies targeting muscle and metabolic health, whereas their application as microbiome-targeted cognitive interventions remains investigational. Future clinical trials incorporating microbiome profiling, metabolomics, gut hormone measurements, and validated cognitive assessments are required before specific recommendations can be established.

## 10 Limitations of current evidence

Despite increasing interest in the potential interactions between whey protein supplementation, RT, gut microbiota, and gut–brain communication, several important limitations restrict the interpretation of current findings. The most critical limitation is the near absence of controlled human studies simultaneously evaluating whey protein supplementation, RT, gut microbiota alterations, microbial metabolites, and cognitive outcomes within the same intervention framework. Consequently, many proposed microbiota-mediated gut–brain mechanisms remain based on indirect evidence derived from separate research areas, including whey protein studies, RT interventions, animal models, and microbiome investigations. A major limitation of the existing literature is the heterogeneity of study designs. Many investigations involve relatively small sample sizes, short intervention durations, and variable participant characteristics, reducing statistical power and limiting the ability to determine clinically meaningful effects. Short-term interventions frequently demonstrate modest or inconsistent changes in microbial composition, making it difficult to establish whether observed alterations represent transient adaptations or long-term biological remodeling. Considerable variability also exists in intervention characteristics, including whey protein source, processing method, dosage, supplementation duration, dietary background, and exercise protocols. In particular, evidence specifically related to RT remains limited, as many studies cited in the broader exercise literature involve endurance or mixed exercise modalities. Therefore, extrapolation from general exercise findings to RT-specific microbiota and gut–brain effects should be interpreted cautiously. Methodological differences in microbiome analysis further complicate comparisons between studies. Variations in sequencing platforms, bioinformatic pipelines, taxonomic classification methods, and reporting standards may contribute to inconsistent findings. Moreover, many studies focus primarily on microbial composition, whereas functional assessments involving metagenomics, metabolomics, microbial metabolites, and host-response markers remain relatively uncommon. This limitation is particularly important because changes in microbial taxa do not necessarily indicate functional alterations relevant to gut–brain communication. The generalizability of current findings is also restricted by population limitations. A substantial proportion of studies involve athletes, healthy young adults, or preclinical models, whereas evidence from older adults, females, and clinical populations remains comparatively limited. Sex-specific responses are particularly understudied, despite potential differences in hormonal status, metabolism, microbiota composition, and exercise adaptation. Cognitive and neurological outcomes represent another major knowledge gap. Although some studies report improvements in executive function, sleep-related parameters, or neurophysiological markers following whey protein or RT interventions, these outcomes are inconsistent and rarely accompanied by direct microbiome measurements. Therefore, claims regarding microbiota-mediated cognitive benefits should be considered hypothesis-generating rather than established mechanisms. Finally, important confounding factors, including habitual dietary intake, medication use, lifestyle behaviors, exercise adherence, baseline microbiota variability, and individual metabolic characteristics, are not consistently controlled across studies. Addressing these limitations will require large-scale, longitudinal, standardized clinical trials incorporating microbiome sequencing, metabolomics, gut hormone profiling, inflammatory biomarkers, and validated cognitive assessments to clarify whether whey protein and RT can meaningfully influence gut–brain communication.

## 11 Future perspectives and research directions

Future research should prioritize the development of rigorous experimental frameworks to clarify the relationship between whey protein supplementation, RT, gut microbiota, and gut–brain communication. A major priority is the establishment of well-designed randomized controlled trials that simultaneously evaluate whey protein intake, RT-specific protocols, microbiome alterations, microbial metabolites, and cognitive outcomes within the same study population. Such integrated designs are essential to distinguish direct microbiota-mediated effects from parallel physiological adaptations related to exercise, nutrition, inflammation, or metabolic regulation. Future clinical investigations should adopt standardized approaches regarding protein source, dosage, supplementation duration, exercise modality, intensity, and adherence monitoring. Particular attention should be given to RT-specific interventions, as much of the current evidence is derived from endurance or mixed exercise studies and cannot be directly generalized to resistance-based programs. Longitudinal studies are also required to determine whether microbial changes observed following short-term interventions represent transient responses or stable adaptations associated with long-term health outcomes. The integration of multi-omics approaches, including metagenomics, metabolomics, transcriptomics, and proteomics, will be critical for advancing beyond descriptive analyses of microbial composition. Future studies should focus on functional microbial outputs, including SCFAs, secondary bile acids, indole derivatives, neurotransmitter-related metabolites, and inflammatory mediators, to determine whether these factors serve as measurable intermediaries within gut–brain communication pathways. Another important research direction involves identifying sources of interindividual variability in response to whey protein and exercise. Future studies should incorporate baseline microbiota characteristics, dietary patterns, genetic factors, age, sex, hormonal status, and health conditions to determine whether specific populations exhibit differential responsiveness. Sex-disaggregated analyses should be incorporated, as current evidence remains insufficient regarding potential differences between males and females. The potential interaction between whey protein and other dietary components, including prebiotics, probiotics, fermented foods, and bioactive compounds, also warrants further investigation. However, future trials should use appropriate factorial designs to determine the independent and combined contributions of each component rather than attributing effects to complex multi-ingredient interventions. Similarly, the influence of protein timing relative to exercise and circadian rhythms remains an emerging area requiring systematic evaluation. Finally, future research should expand beyond healthy athletes and younger populations by including older adults, individuals with metabolic dysfunction, GI disorders, and neurological conditions. Nevertheless, translation into clinical practice should depend on the confirmation of causal mechanisms and reproducible benefits in well-controlled human studies. Collectively, these research directions will help determine whether whey protein and RT represent effective strategies for modifying microbiota-mediated gut–brain communication or whether observed associations primarily reflect broader metabolic and lifestyle-related adaptations.

## 12 Conclusion

The current body of evidence suggests that whey protein supplementation combined with RT may influence gut microbiota composition, GI physiology, metabolic regulation, and gut–brain communication; however, the extent to which microbiota-mediated mechanisms contribute to these outcomes remains incompletely established. Although whey protein and RT individually exert biological effects on host metabolism and microbial ecology, direct evidence demonstrating that their combined administration induces cognitive benefits through microbiota-dependent pathways is currently limited. Most available findings are derived from separate lines of evidence, including whey protein interventions, exercise studies involving different modalities, and preclinical investigations, rather than from controlled human trials simultaneously assessing microbiota alterations, microbial metabolites, and cognitive outcomes. Whey protein supplementation appears capable of modifying gut microbial composition and microbial metabolic activity, but these responses are highly context dependent and influenced by protein source, dosage, dietary background, intervention duration, and individual microbiome characteristics. Similarly, exercise-induced microbial adaptations vary according to exercise modality, intensity, and duration, with RT-specific evidence remaining relatively scarce compared with aerobic or mixed RT interventions. Therefore, although increased microbial diversity, SCFA production, and improved intestinal barrier-related signaling represent plausible mechanisms, their contribution to gut–brain communication requires further validation through well-controlled mechanistic studies. From a gut–brain axis perspective, whey protein and RT may interact with neuroendocrine, immune, and metabolic pathways involving microbial metabolites, gut hormones, inflammatory signaling, and neurotrophic mechanisms. These pathways provide a biologically plausible framework linking nutritional and RT interventions with cognitive health; nevertheless, current evidence does not support definitive conclusions regarding synergistic effects of whey protein and RT on cognition. Existing clinical studies have reported variable cognitive outcomes, with some demonstrating potential improvements in specific domains, whereas others have failed to identify significant benefits or clear interaction effects between protein supplementation and exercise. Future research should prioritize long-term, adequately powered randomized controlled trials that directly integrate microbiome profiling, metabolomic analyses, and validated cognitive assessments. Greater attention should also be directed toward sex-specific responses, clinical populations, baseline microbiota variability, dietary control, and standardized RT protocols. Such investigations will be essential to determine whether microbiota-targeted nutritional and exercise strategies can be translated into effective approaches for supporting GI function, metabolic health, and cognitive resilience.
